# Targeting claudin‐4 enhances chemosensitivity of pancreatic ductal carcinomas

**DOI:** 10.1002/cam4.2547

**Published:** 2019-09-09

**Authors:** Takamitsu Sasaki, Rina Fujiwara‐Tani, Shingo Kishi, Shiori Mori, Yi Luo, Hitoshi Ohmori, Isao Kawahara, Kei Goto, Yukiko Nishiguchi, Takuya Mori, Masayuki Sho, Masuo Kondo, Hiroki Kuniyasu

**Affiliations:** ^1^ Department of Molecular Pathology Nara Medical University Kashihara Nara Japan; ^2^ Jiangsu Province Key Laboratory of Neuroregeneration Nantong University Nantong Jiangsu China; ^3^ Department of Surgery Nara Medical University Kashihara Nara Japan; ^4^ Drug Innovation Center Graduate School of Pharmaceutical Sciences Osaka University Suita Osaka Japan

**Keywords:** claudin, tight junction

## Abstract

Claudin (CLDN) family comprises of protein that form a tight junction, and is involved in regulating polarity and differentiation of cells. Here, we aimed to investigate the effects of inhibiting CLDN4 in pancreatic ductal carcinomas (PDC). We first examined 91 cases of human PDC by immunohistochemistry and found that CLDN4 expression was correlated with tumor invasion, nodal metastasis, and distant metastasis. Anti‐CLDN4 extracellular domain antibody, previously established by us (4D3), inhibited the proliferation of MIA‐PaCa‐2 PDC cells and increased intracellular 5‐fluorouracil (5‐FU) concentration with lowering transepithelial electrical resistance. Concurrent treatment of 5‐FU and 4D3 resulted in synergistic inhibition of growth of MIA‐PaCa‐2 cells in nude mice. In addition, MIA‐PaCa‐2 cell tumors treated with full‐dose folfirinox (FFX) decreased tumor diameters to 50%; however, 60% of mice were dead from adverse effects. In contrast, half‐dose FFX concomitant with 4D3 treatment decreased tumors equivalent to full‐dose FFX, but without the adverse effects. These findings suggest that targeting CLDN4 might increase the effectiveness and safety of anticancer drug therapy in PDC.

## Graphical abstract

Inhibition of tight junction by anti‐claudin‐4 antibody (4D3) decreases barrier function in cancer, enhances intratumoral permeation and the antitumor effect of anticancer agents. 4D3 treatment with half‐dose folfirinox (FFX) results in decreased tumors, equivalent to that achieved with full‐dose FFX without the adverse effects of full‐dose FFX in a mouse model.

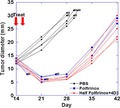

## 1 INTRODUCTION

Pancreatic ductal carcinoma (PDC) is the fourth leading cause of death by cancer in Japan, and it shows long‐term increase in incidence.^1^ The clinical features associated with PDC are as follows: it has poor specific symptoms and is difficult to detect early; it poses a strong physical burden to patients and has to be treated surgically; incidence of metastasis and recurrence is high; and the effectiveness of chemotherapy is unsatisfactory.^2^, ^3^, ^4^ It's overall 5‐year survival is approximately 8% for all stages combined; however, the majority of patients present with stage IV disease at diagnosis and show an overall 5‐year survival of 3%.^2^

As part of chemotherapy, gemcitabine (GEM) or S‐1 is frequently used as the first‐line drug; however, in most cases it causes drug resistance. To increase effectiveness, folfirinox (FFX; 5‐fluorouracil [5‐FU] + folinate calcium [1‐LV] + irinotecan [CPT‐11] + oxaliplatin [L‐OHP]) has been devised as a regimen that is concurrently applied as FOLFOX (5‐FU + l‐LV + L‐OHP) and FOLFIRI (5‐FU + l‐LV + CPT‐11) for PDC. Folfirinox is superior to metastatic PDC than GEM monotherapy.^5^ However, although improvement in efficacy is confirmed, there are many cases where it is difficult to complete the protocol due to severe adverse effects of FFX such as bone marrow suppression and mucosal damage.^6^, ^7^

Tight junction is one of the intercellular adhesion structures and controls the traffic of substances between cells.^8^ In the mucosal epithelium, tight junction acts as a barrier or fence between the lumen of the gastrointestinal tract and the mucosa covered by the epithelium.^8^, ^9^ This function prevents the transfer of harmful substances from the gastrointestinal tract to the mucous membrane and outflow of physiologically active substances from the mucosa into the digestive tract. On the other hand, in cancer tissues, tight junctions also play a role in maintaining the cancer microenvironment by inhibiting the invasion of anticancer drugs into tumor tissues, and promoting intratumoral retention of growth factors.^10^, ^11^, ^12^

CLDN4, belonging to the claudin (CLDN) protein family, is a major constituent protein of the tight junction. CLDN forms a family of 27 different proteins with very similar structures.^8^, ^9^, ^13^ CLDN4 exhibits high expression in epithelial tissues, with expression also seen in epithelial malignant tumors.^14^, ^15^, ^16^, ^17^ We have been studying its potential application in cancer therapy by preparing antibodies specifically recognizing the extracellular domain of CLDN4.^12^ As a result, it was found that inhibition of tight junction by anti‐CLDN4 extracellular domain antibody (4D3) decreases barrier function in cancer, promotes intratumoral migration of anticancer agent, and enhances its antitumor effect.^10^, ^11^, ^12^

In this study, we investigated the effect of 4D3 on PDC and assessed its safety and efficacy in combination treatment with FFX.

## 2 MATERIALS AND METHODS

### 2.1 Surgical specimens

We reviewed the pathological diagnosis and clinical data of 91 patients diagnosed with PDC in the Department of Molecular Pathology, Nara Medical University from 2004 to 2015. As written informed consent was not obtained, any identifying information was removed from the samples prior to analysis, in order to ensure strict privacy protection (unlinkable anonymization).

All procedures were performed in accordance with the Ethical Guidelines for Human Genome/Gene Research issued by the Japanese Government and were approved by the Ethics Committee of Nara Medical University (Approval Number 937).

### 2.2 Cell lines

MIA‐PaCa‐2 human PDC cell line was purchased from Dainihon Pharmaceutical Co. Cells were cultured in Dulbecco's modified Eagle's medium supplemented with 10% fetal bovine serum at 37°C in 5% CO_2_. Cell growth was assessed using a tetrazolium (MTT) dye assay, as previously described.^18^

### 2.3 Antibody and reagents

The anti‐human CLDN4 extracellular domain antibody 4D3 was developed by immunizing rats with a plasmid vector encoding human CLDN4.^12^ The B cells isolated from the rats with an increased serum titer of anti‐hCLDN4 antibody are fused with myeloma cells (P3U1) to result in the production of hybridoma cells. The anti‐human CLDN1 extracellular domain antibody 2C1 was also developed by the same method.^19^ The 5‐FU, CPT‐11, and L‐OHP were purchased from Wako Pure Chemical Corp. Ltd., Osaka, Japan. Calcium folinate was purchased from TCI Chemicals Inc. (Tokyo, Japan).

### 2.4 Sphere assay

MIA‐PaCa‐2 cells (5 × 10^4^) were grown in stem cell medium (Sigma) in 6‐well bacteriological grade plates (Gibco) and incubated at 37°C in 5% CO_2_. After 5 hours, the cells were treated with 4D3 or 2C1 for 24 hours.^11^

### 2.5 Animals

BALB/c nude mice (4 weeks old, male) were purchased from SLC Japan. The mice were maintained according to the institutional guidelines approved by the Committee for Animal Experimentation of Nara Medical University, in accordance with the current regulations and standards of the Ministry of Health, Labor, and Welfare.

To establish a subcutaneous tumor model, MIA‐PaCa‐2 cells (1 × 10^7^) were inoculated subcutaneously into the scapular tissues of nude mice. Then, with five mice in each group, 5‐FU (10 mg/kg body weight [BW]) and/or 4D3 (1 mg/kg BW, diluted with saline) were injected into the peritoneal cavity simultaneously on Days 1, 3, and 7. Tumor size was monitored weekly.

For FFX treatment, MIA‐PaCa‐2 cells (1 × 10^7^) were inoculated subcutaneously into the scapular tissues of nude mice. After tumor growth, anticancer drugs and/or 4D3 were administered on Day 14 and 16. With five mice in each group, mice were administered with full‐dose FFX (1‐LV 100 mg/kg BW, L‐OHP 5 mg/kg BW, CPT‐11 50 mg/kg BW and 5‐FU 50 mg/kg in 10% dimethyl sulfoxide [DMSO])^20^ or half‐dose FFX (half dose of each drug) + 4D3 (1 mg/kg BW), injected into the peritoneal cavity. Tumor size was monitored weekly. According to the institutional humane endpoint for animal experiments, moribund mice were euthanized.

For blood analysis, 100 μL of blood was drawn from the tail vein. Red and white blood corpuscles (RBC and WBC, respectively) were counted by an automated hematology analyzer (Sysmex Corp.).

### 2.6 Immunohistochemistry

Consecutive 4‐μm sections were immunohistochemically stained using 0.2 µg/mL 4D3 and a previously described immunoperoxidase technique.^21^ Secondary antibodies (Medical and Biological Laboratories) were used at a concentration of 0.2 µg/mL. Tissue sections were color‐developed with diamine benzidine hydrochloride (DAKO) and counterstained with Meyer's hematoxylin (Sigma). We counted immunopositive cells at the cytoplasmic membrane. Staining strength was scored from 0 to 3 (a score of 1 was used to describe the expression level in normal pancreatic duct epithelium). The staining index was calculated as the staining strength score multiplied by the staining area (%). For a negative control, non‐immunized rat immunoglobulin G (IgG) (Santa‐Cruz Biotechnology) was used as the primary antibody.

### 2.7 Immunoblot analysis

Whole‐cell lysates were prepared as previously described.^22^ Lysates (20 μg) were subjected to immunoblot analysis using sodium dodecyl sulfate‐polyacrylamide gel electrophoresis (12.5%), followed by electrotransfer onto nitrocellulose filters. The filters were incubated with primary antibodies, followed by peroxidase‐conjugated IgG antibodies (Medical and Biological Laboratories). Anti‐tubulin antibody was used to assess the levels of protein loaded per lane (Oncogene Research Products). The immune complex was visualized using an Enhanced Chemiluminescence Western‐blot detection system (Amersham). Antibody for caspase‐3 (Abcam plc) was used as primary antibodies.

### 2.8 Transepithelial electroresistance

A tight junction monitoring system, cellZscope (Fujifilm) was employed to measure the transepithelial electroresistance (TER) of the MIA‐PaCa‐2 cells (1 × 10^5^ cells, seeded onto the insert well to form multiple cell layers). For a positive control, cytochalasin B (CCB; 10 μmol/L; Wako) was used to impair tight junction.

### 2.9 Enzyme‐linked immunosorbent assay

Enzyme‐linked immunosorbent assay (ELISA) kits were used to measure the concentrations of hypoxia inducible factor (HIF)‐1α (Cell Biolabs, Inc), 5‐FU (anti‐5‐Fluorouracil antibody‐derived ELISA; MABEL Inc), ALT (BioVision, Inc) and amylase (MaxDiscovery amylase assay kit; Perkin Elmer) according to the manufacturers' instructions.

### 2.10 Statistical analysis

Statistical significance was calculated using two‐tailed Fisher's exact, chi‐square, and unpaired Mann‐Whitney tests with InStat software (GraphPad). Survival was calculated by Kaplan‐Meier test. Statistical significance was defined as a two‐sided *p*‐value of <.05.

## 3 RESULTS

### 3.1 Expression of CLDN4 in human PDC

**Figure 1:**
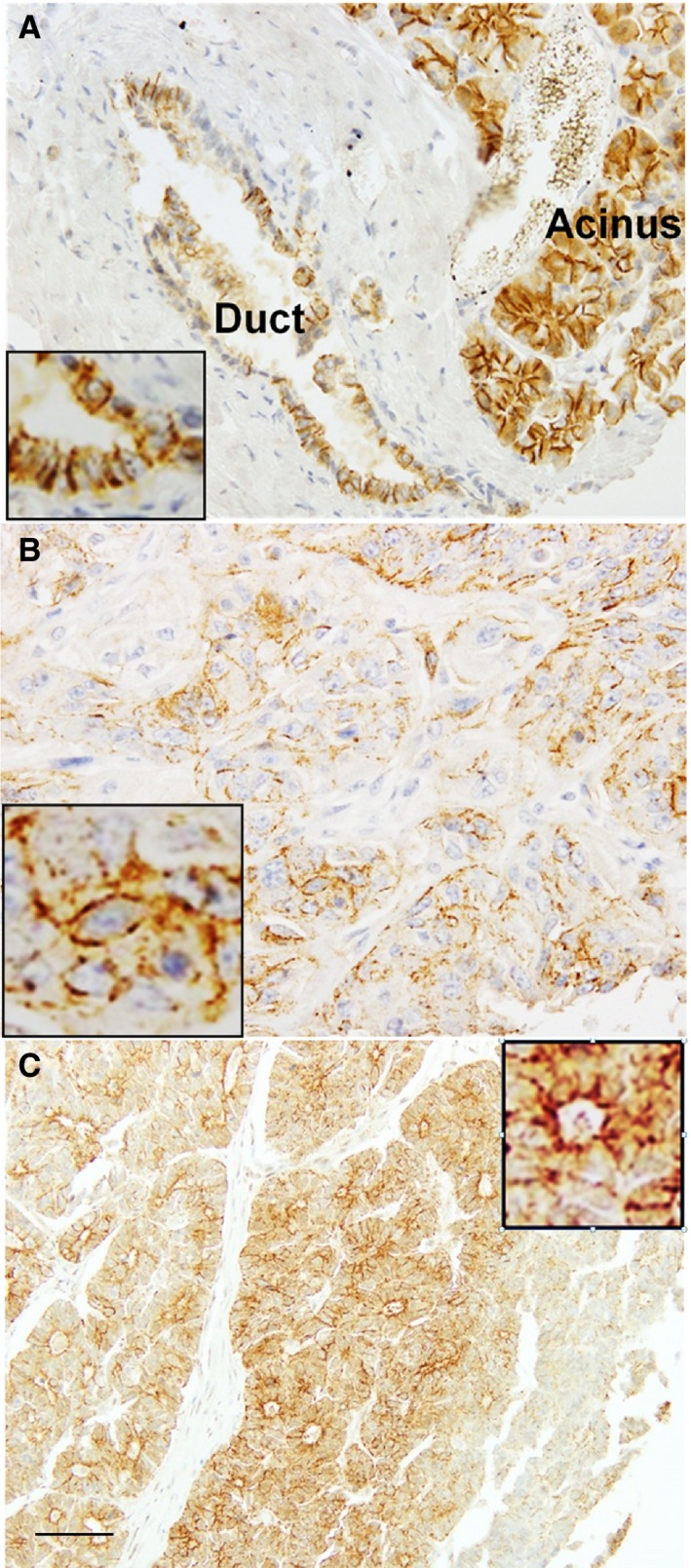
CLDN4 expression in pancreatic ductal carcinoma (PDC). A, An immunohistochemical evaluation identified CLDN4 expression at the cytoplasmic membrane of the normal pancreatic duct epithelia and acinus. B, CLDN4 expression in a PDC case of pT3/pN0/pM0. C, CLDN4 expression in a PDC case of pT3/pN1/pM1. Inset of each panel, high magnification image. Bar, 100 μm

CLDN4 expression was examined by immunohistochemistry in 91 PDCs (Figure 1). In non‐cancerous pancreatic tissues, CLDN4 was located at the cytoplasmic membrane in pancreatic duct epithelium and acinar cell (Figure 1A). Similarly, the PDC cells showed CLDN4 expression at the cytoplasmic membrane.

We next compared the CLDN4 expression with clinicopathological parameters in the 91 PDC cases (Table 1). CLDN4 expression index was associated with primary tumor (pT), nodal metastasis (pN), distant metastasis (pM) and pathological stage, but not with histological grade.

**Table 1:** Expression of CLDN4 in 91 pancreatic ductal carcinomas

Parameter	n	CLDN4 expression index^a^ (mean ± SD)	*P* b
Sex			
Male	54	202 ± 117	
Female	37	210 ± 91	NS
Age			
~50 y	37	199 ± 91	
~51 y	54	209 ± 125	NS
Histological grade^c^			
G1	27	209 ± 78	
G2	22	184 ± 80	
G3	42	213 ± 84	NS
Tumor invasion (pT)^c^			
pT1‐pT2	30	183 ± 71	
pT3‐pT4	61	216 ± 70	<.05
Nodal metastasis (pN)^c^			
pN0	79	198 ± 80	
pN1‐pN2	12	247 ± 52	<.05
Distant metastasis (pM)^c^			
pM0	88	202 ± 84	
pM1	3	300 ± 1	<.05
Pathological stage^c^			
I	26	172 ± 92	
II	51	208 ± 79	
III‐IV	14	254 ± 52	<.01

Abbreviation: CLDN, claudin.

a The staining index was calculated as the staining strength score (0‐3) multiplied by the staining area (%).

b *P* value was calculated by student *t* test.

c Clinicopathological parameters were classified according to AJCC.^29^ pT1, tumor limited to the pancreas, ≤2 cm in greatest dimension; pT2, tumor limited to the pancreas, >2 cm in greatest dimension; pT3, tumor extends beyond the pancreas but without involvement of the celiac axis or the superior mesenteric artery; pT4, tumor involves the celiac axis or the superior mesenteric artery (unresectable primary tumor); pN0, no regional lymph node metastasis; pN1, regional lymph node metastasis; pM0, no distant metastasis; pM1, distant metastasis; stage I, pT1 or pT2 and pN0; stage II, pT1‐3 and pN1 or pT3 and pN0; stage III, pT4 and any pN; stage IV, any pT, any pN and pM1.

### 3.2 Effect of 4D3 in human PDC cell line

**Figure 2:**
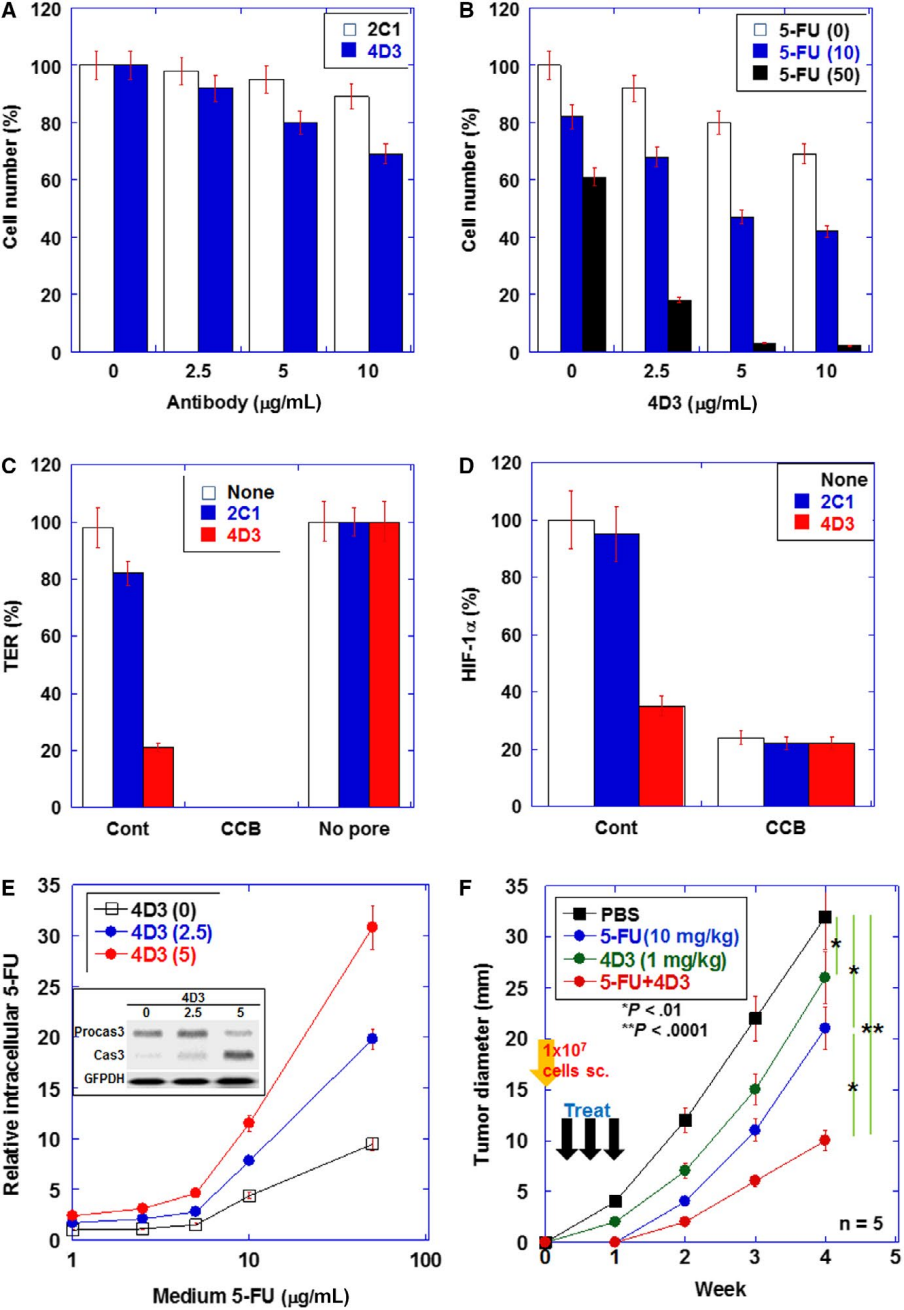
Effects induced by the 4D3 antibody in MIA‐PaCa‐2 human pancreatic ductal carcinoma (PDC) cells in vitro and in vivo. A, Comparison of growth inhibitory effects between anti‐CLDN4 antibody (4D3) and anti‐CLDN1 (2C1) in MIA‐PaCa‐2 human PDC cells. B, The combined effects of 5‐FU and 4D3 on cell proliferation. C, The transepithelial electrical resistance (TER) of MIA‐PaCa‐2 cells treated with 4D3 or 2C1 was measured. Cytochalasin B (CCB) was used to dissociate cells (negative control). D, Hypoxia inducible factor (HIF)‐1α protein levels were examined by enzyme‐linked immunosorbent assay (ELISA) in spheres of MIA‐PaCa‐2 cells treated with 4D3 or 2C1. CCB was used to dissociate cells. E, The intracellular 5‐FU concentration was measured by ELISA in cells with or without 4D3 treatment. Inset: protein levels of procasepase‐3 (Procas3) and caspase‐3 (Cas3) in MIA‐PaCa‐2 cells treated with 5‐FU (50 µg/mL) and 4D3. F, Effect of concurrent treatment of 5‐FU and 4D3 on growth of MIA‐PaCa‐2 cells in nude mice. In nude mice, MIA‐PaCa‐2 tumors were treated with 5‐FU (5 mg/kg body weight [BW]) and/or 4D3 (1 mg/kg BW) on Day 1, 3, and 7. The SD was calculated from three independent trials or five mice

We previously established 4D3 antibody for targeting CLDN4 in cancer cells.^12^ MIA‐PaCa‐2 cells were treated with 4D3 and compared with those treated with 2C1^19^ (Figure 2A). 2C1 did not show significant growth inhibition of MIA‐PaCa‐2 cells, whereas 4D3 showed significant growth inhibition in a dose‐dependent manner. 4D3 enhanced 5‐FU‐induced growth inhibition in a dose‐dependent manner at each 5‐FU concentration (Figure 2B).

To examine the damage in tight junction by 4D3, TER was measured (Figure 2C). 2C1 showed 19% decrease in TER, whereas 4D3 induced 79% decrease in TER. Since impairment of tight junction results in abrogation of the intratumoral microenvironment,^12^ we assessed the microenvironment by measuring HIF‐1α production (Figure 2D). Our results showed that 4D3, but not 2C1, decreased HIF‐1α production in spheres of MIA‐PaCa‐2 cells, suggesting that 4D3 induced damage of the tight junction.

Consistent with enhanced drug penetration into tumor tissues owing to impaired tight junction,^12^ intracellular 5‐FU levels were found to increase in 4D3‐treated MIA‐PaCa‐2 cells in a dose‐dependent manner (Figure 2E). Protein levels of procasepase‐3 (Procas3) and caspase‐3 (Cas3) were examined in MIA‐PaCa‐2 cells treated with 5‐FU (50 μg/mL) and 4D3 for assessing apoptosis (Figure 2E inset). Mature caspase‐3 levels were increased in a dose‐dependent manner with 4D3. We then examined the antitumoral effect of concurrent treatment with 4D3 and 5‐FU (Figure 2F). Treatment of the subcutaneous tumors in MIA‐PaCa‐2 cell line with 4D3 alone and 5‐FU alone resulted in growth inhibition by 22% and 34%, respectively, while the simultaneous treatment with both showed growth inhibition by 69%, which was thought to be a synergistic effect.

### 3.3 Effect of 4D3 on antitumoral effects of L‐OHP, CPT‐11, and 5‐FU

**Figure 3:**
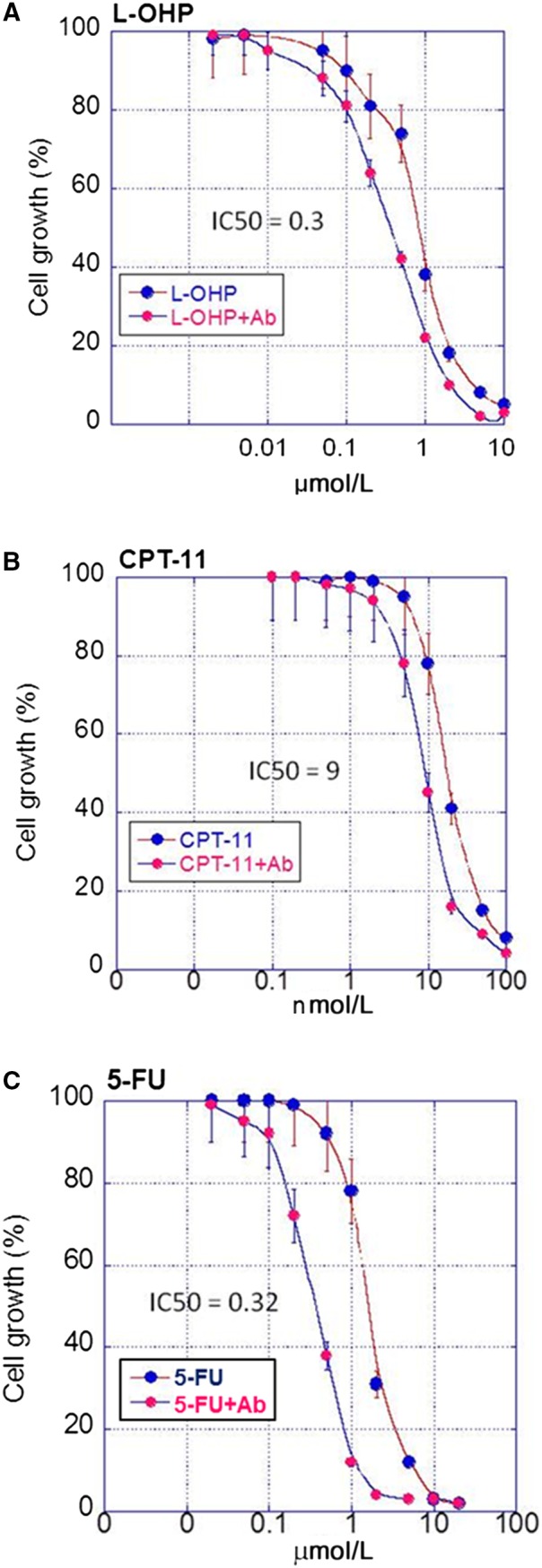
The combined effects of 4D3 and three drugs of folfirinox on cell proliferation. MIA‐PaCa‐2 cells were treated with (A) oxaliplatin (L‐OHP), (B) irinotecan (CPT‐11) and (C) 5‐fluorouracil (5‐FU) with or without 4D3. The SD was calculated from three independent trials. IC50, 50% inhibitory concentration (μmol/L)

Next, we examined the antitumor effect of concurrent treatment of 4D3 with three anticancer drugs of FFX. As shown in Figure 3, the inhibitory concentration (IC) 50 of L‐OHP decreased by 62% from 0.8 to 0.3 by concurrent treatment with 4D3 (Figure 3A). Similarly, the IC50 of CPT‐11 and 5‐FU decreased by 22% (from 11.6 to 9; Figure 3B) and 81% (from 1.7 to 0.32 for 5‐FU; Figure 3C), respectively, by concurrent treatment with 4D3. Thus, the simultaneous treatment of 4D3 with each of the anticancer drugs promoted the antitumor effect.

### 3.4 Effect of 4D3 on antitumoral effect of FFX

**Figure 4:**
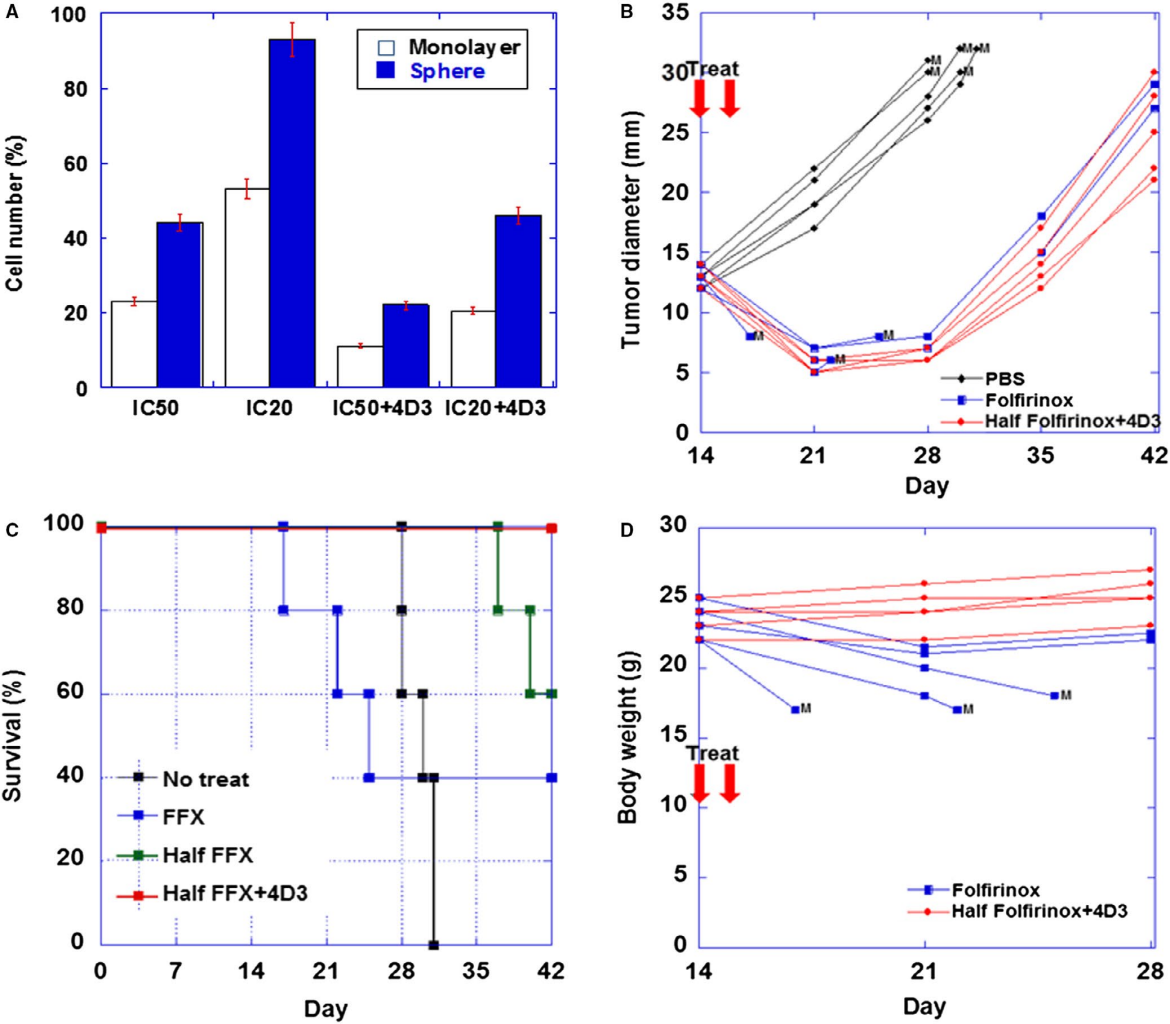
Effect of 4D3 on tumor inhibitory effect of folfirinox (FFX) in in vitro and in vivo. A, MIA‐PaCa‐2 cells were treated concurrently with oxaliplatin (L‐OHP), irinotecan (CPT‐11), and 5‐fluorouracil (5‐FU) with or without 4D3 in monolayer or sphere culture. The SD was calculated from three independent trials. B, Diameter of the subcutaneous tumor of MIA‐PaCa‐2 cells in individual mouse treated with phosphate buffered saline (PBS control), full‐dose FFX (calcium folinate 100 mg/kg, L‐OHP 5 mg/kg, CPT‐11 50 mg/kg, and 5‐FU 50 mg/kg, ip) or half‐dose FFX (calcium folinate 50 mg/kg, L‐OHP 2.5 mg/kg, CPT‐11 25 mg/kg, and 5‐FU 25 mg/kg, ip) plus 4D3 (1 mg/kg BW, ip). M, moribund (euthanized). C, Body weight of individual mouse treated with full‐dose FFX or half‐dose FFX. M, moribund (euthanized). D, Survivals of mice treated with PBS (No treat), full‐dose FFX (FFX), half‐dose FFX (Half FFX), or half‐dose FFX plus 4D3 (Half FFX + 4D3)

We examined the antitumor effect of 4D3 on MIA‐PaCa‐2 cells treated with the IC50 or IC20 for the three anticancer drugs of FFX (Figure 4A). In monolayer culture, the antitumor effect of IC50 and IC20 doses of the drugs when treated in combination with 4D3 was promoted by 1.2 times and 1.7 times, respectively. Similarly, in sphere culture, the antitumor effect of the IC50 and IC20 doses was promoted by 1.4 times and 6.5 times, respectively.

### 3.5 Effect of 4D3 on the antitumoral and side effects seen in FFX‐treated mice

Subcutaneous tumors of MIA‐PaCa‐2 cells in nude mice were treated with full‐dose FFX, half‐dose FFX plus 4D3 or vehicle (10% DMSO) at 2 weeks after inoculation (Figure 4B). In each of the five untreated mice, the tumor increased to approximately 3 cm in diameter at approximately 4 weeks, and the mice became moribund and were euthanized. In contrast, in the full dose group and the half dose + 4D3 group, the tumors were reduced to about 51% and 48%, respectively, 1 week after treatment without regrowth for another week. However, three of five mice in the full dose group became moribund and were euthanized. In the surviving mice of the treated groups, the tumors re‐grew to 2‐3 cm; however, the survival periods were more than 2 weeks in comparison with untreated mice.

As shown in Table 2, the half dose FFX + 4D3 group showed smaller tumor diameter than that in the half dose FFX group. Untreated mice showed the largest tumor diameter and higher BW due to tumors. The full dose FFX group showed small tumor diameter because of the early death by drug toxicity. Overall survivals of mice treated with PBS (No treat), full‐dose FFX (FFX), half‐dose FFX (Half FFX) or half‐dose FFX plus 4D3 (Half FFX + 4D3) were compared (Figure 4C). All mice of the half dose FFX + 4D3 group survived during the observation period, whereas the half dose FFX group showed two mice were dead from tumor. FFX group, two mice that survived from anticancer drug toxicity survived during the observation period.

**Table 2:** Effect of folfirinox and 4D3

	No treat	Folfirinox		
		Full dose	Half dose	Half dose + 4D3
n	5	5	5	5
Survival	0/5	2/5	3/5	5/5
Tumor diameter (mm)^a^	29.7 ± 5.4	17.6 ± 12.8	31.4 ± 7.0	24.5 ± 8.2^b^
Body weight (g)^a^	28.1 ± 2.5	18.4 ± 2.2	23.7 ± 2.8	23.3 ± 1.8

a Tumor diameter and body weight were measured at death or at the end of observation.

b Tumor diameter in mice treated with half dose folfirinox + 4D3 was significantly smaller than those in mice treated with half dose folfirinox (*P* < .0001, Student *t* test with Bonferroni correction).

Mouse BW change after treatment was examined (Figure 4D). Weight loss was not observed in half dose + 4D3 group, whereas in the full dose group, weight loss was observed in all mice, and the three dying animals showed drastic decline. On Day 21, RBC, WBC, ALT, and amylase in the bloods were examined (Table 3). Mice treated with full dose FFX showed decreased RBC and WBC counts, and increase of ALT in comparison with those in untreated or half dose FFX‐treated mice. Moreover, RBC, WBC, ALT, and amylase in the mice treated with half dose FFX plus 4D3 were not different from those in untreated mice.

**Table 3:** Effect of folfirinox on blood corpuscles and liver function

	n	No treat	Folfirinox		
			Full dose	Half dose	Half dose + 4D3
RBC (×10^4^)	5	1030 ± 74	684 ± 65^a^	968 ± 62	967 ± 72
WBC (×10^2^)	5	24.5 ± 7.2	16.2 ± 5.8^a^	22.8 ± 7.0	23.7 ± 8
ALT (U/L)	5	34 ± 4.1	108 ± 18^a^	36 ± 4.4	35 ± 3.8
Amylase (U/L)	5	527 ± 38	524 ± 74^a^	510 ± 68	505 ± 76

Abbreviations: ALT, alanine aminotransferase; RBC, red blood corpuscle; WBC, white blood corpuscle.

a Significantly different among four groups (*P* < .05, by AOVA).

## 4 DISCUSSION

In this study, we have shown that inhibition of tight junction by anti‐CLDN4 antibody 4D3 promoted the intratumoral invasion of anticancer drugs and enhanced their antitumor effects. Furthermore, by using 4D3 and only half‐dose of FFX, it was shown that it is possible to obtain an antitumor effect equivalent to the full‐dose FFX, while reducing the associated adverse effects.

Our data showed that 4D3 abrogated intratumoral microenvironment; decrease of HIF‐1α suggests that hypoxic circumstance might become normoxic by impairing the barrier for oxygen diffusion. Abrogation of the barrier by 4D3 also induced intratumoral permeation of 5‐FU, which enhanced 5‐FU‐induced apoptosis.

Since Conroy et al have reported that FFX shows superiority to GEM monotherapy against metastatic pancreatic cancer, FFX has attracted attention as a treatment protocol for pancreatic cancer.^5^ Patients with locally advanced pancreatic cancer treated with FFX show a median overall survival of 24.2 months, which is longer than that reported with GEM (6‐13 months).^23^ Currently, nab‐paclitaxel and FFX have equivalent effect of prolonging survival time.^24^ FFX and GEM plus nab‐paclitaxel are now considered standard first line treatment options in metastatic pancreatic cancer.^2^, ^25^, ^26^ However, FFX has been reported to have a stronger adverse effect than nab‐paclitaxel.^24^

In the present study, tumors shrunk by 80% upon administration of FFX twice. This is a significant antitumor effect compared with 5‐FU monotherapy (data not shown). However, three of five mice became moribund in 1 week after the administration of FFX twice, and advanced anemia and diarrhea were observed. Thus, high antitumor effect of FFX and a strong adverse effect were also observed in the mouse model. Leucopenia, anemia, and gastrointestinal symptoms such as nausea and diarrhea, are reported in patients administered with FFX,^6^, ^7^ which was similar in our animal model.

In contrast, when the antibody was used in combination with half‐dose FFX, the antitumoral effect was 80% after two treatments, which was equivalent to that of the full‐dose FFX. Importantly, with the combination therapy of the half‐dose FFX and 4D3, no relevant adverse effects were observed in all mice, and none of the mice became moribund. Effect of low dose FFX has been tried clinically, in which the balance between maintenance of therapeutic effect and reduction of adverse events is a problem. Chllamma et al have reported that the progression‐free survival in the locally advanced PDC patients is 11.1 months with full‐dose FFX, whereas it is 10.4 months with modified dose FFX.^27^ However, grade 3/4 hematologic adverse events are observed in nearly half of patients treated with modified dose of FFX.^27^ Our trial of half‐dose FFX using the anti‐CLDN4 antibody 4D3 is considered to be an effective way to alleviate the adverse effects, while maintaining the therapeutic effect of FFX.

Our data show the effect of 4D3 are based on increase of permeation of anti‐cancer agents, impairment of tumor microenvironment and the direct damage on tumor cells with antibody‐dependent cellular cytotoxicity.^10^, ^11^, ^12^ Since our study is based on a mouse model, judgment on the adverse effects cannot be directly extrapolated to humans. Anti‐CLDN4 antibody 4D3 promotes antitumor effects of anticancer agents without relevant adverse events in other animal experiments.^11^, ^12^ CLDN4 was located at the cytoplasmic membrane in pancreatic duct epithelium and acinar cell as well as PDC cells. However, mice treated with 4D3 did not show any elevation of blood amylase. The findings suggest that 4D3 might not evoke pancreatitis. In the study of antibody biodistribution using mouse CLDN4 antibody (5D12), the antibody distribution in pancreatic tissue is not different from the control antibody.^28^ The findings suggest that anti‐CLDN4 antibody might not evoke pancreatitis. From these findings, combination use of 4D3 is expected to promote useful antitumor effect for FFX as well as to alleviate adverse events; however, further studies including future clinical research are required to validate our findings.

## CONFLICT OF INTEREST

None declared.

## Data Availability

The data that support the findings of this study are available from the corresponding author upon reasonable request.
